# Inhibition of long non-coding RNA UCA1 by CRISPR/Cas9 attenuated malignant phenotypes of bladder cancer

**DOI:** 10.18632/oncotarget.14176

**Published:** 2016-12-25

**Authors:** Shuai Zhen, Ling Hua, Yun-Hui Liu, Xiao-Min Sun, Meng-Meng Jiang, Wei Chen, Le Zhao, Xu Li

**Affiliations:** ^1^ Center for Translational Medicine, The First Affiliated Hospital, Xi’an Jiaotong University, Xi’an 710061, P.R. China; ^2^ Key Laboratory for Tumor Precision Medicine of Shaanxi Province, The First Affiliated Hospital of Xi’an Jiaotong University, Xi’an 710061, P.R. China; ^3^ Department of Veterinary Medicine, Rongchang Campus of Southwest University, Chongqing 402460, P.R. China; ^4^ Department of Pharmacology and Toxicology, Beijing Institute of Radiation Medicine, Beijing 100850, P.R. China; ^5^ Center for Laboratory Medicine, The First Affiliated Hospital of Xi’an Jiaotong University, Xi’an 710061, P.R. China; ^6^ State Key Laboratory of Cancer Biology, The Fourth Military Medical University, Xi’an 710032, Shaanxi, China

**Keywords:** CRISPR/Cas9, long non-coding RNA, UCA1, bladder cancer

## Abstract

CRISPR/Cas9 is a novel and effective genome editing technique, but its application is not widely expanded to manipulate long non-coding RNA (lncRNA) expression. The lncRNA urothelial carcinoma-associated 1 (UCA1) is upregulated in bladder cancer and promotes the progression of bladder cancer. Here, we design gRNAs specific to UCA1 and construct CRISPR/Cas9 systems targeting UCA1. Single CRISPR/Cas9-UCA1 can effectively inhibit UCA1 expression when transfected into 5637 and T24 bladder cancer cells, while the combined transfection of the two most effective CRISPR/Cas9-UCA1s can generate more satisfied inhibitory effect. CRISPR/Cas9-UCA1s attenuate UCA1 expression via targeted genome-specific DNA cleavage, resulting in the significant inhibition of cell proliferation, migration and invasion *in vitro* and *in vivo*. The mechanisms associated with the inhibitory effect of CRISPR/Cas9-UCA1 on malignant phenotypes of bladder cancer are attributed to the induction of cell cycle arrest at G1 phase, a substantial increase of apoptosis, and an enhanced activity of MMPs. Additionally, urinary UCA1 can be used as a non-invasive diagnostic marker for bladder cancer as revealed by a meta-analysis. Collectively, our data suggest that CRISPR/Cas9 technique can be used to down-modulate lncRNA expression, and urinary UCA1 may be used as a non-invasive marker for diagnosis of bladder cancer.

## INTRODUCTION

In recent year, the emergence of genome-editing techniques bring a great change in genomics from an observational to an experimental science. Through willingly and readily deleting or repressing genomic elements, their function can be examined in a natural setting [1]. Clustered regularly interspaced short palindromic repeats (CRISPR) is identified as a natural bacterial immunity system, promising a cheap, simple and versatile genome editing technique [2]. The system is composed of two parts: a “guide RNA” (gRNA) recognizing a specific location in the genome, and the Cas9 protein binding and using gRNA to locate site-specific DNA sequence, and finally introducing a double strand break. Altering the first 20nt of the gRNA can direct Cas9 nuclease to specific sites, CRISPR/Cas9 therefore provides uniquely flexible and accessible means for genome editing [3, 4, 5, 6]. In view of its specificity, efficiency, simplicity and versatility, the CRISPR/Cas9 technique has achieved numerous successes as a robust genome engineering tool for the treatment of many diseases [7, 8, 9, 10, 11].

It is well known that more than 90% of the human genome is actively transcribed; but only about 2% of the genome encode protein [12], and the rest of the genome encode non-coding RNAs (ncRNAs) including microRNAs and long non-coding RNAs (lncRNAs). LncRNAs are defined as transcripts of more than 200 nucleotides in length with minimal to no protein-coding function [14]. LncRNAs have been recently defined as a new class of regulatory factors that modulate gene expression in both physiological and pathological states [15]. Their biological roles have been increasingly recognized [16, 17].

Although accumulating evidences suggest that lncRNAs such as HOTAR, HOST2 are upregulated and play important roles in cancers [18, 19, 20], functions of overexpressed lncRNA in malignancy diseases remain to be elucidated. One common method for functional study of upregulated lncRNA is RNA inference (RNAi) [21]. However, the nuclear localization of many lncRNAs [22] makes RNAi less effective to achieve robust knocking down efficiency since it is primarily functional in the cytoplasm. Nowadays, the novel genome editing techniques provide better alternatives for inhibiting gene transcription. But their application on modulating lncRNA has not been widely verified.

We have characterized the lncRNA UCA1 from human bladder cancer cell line BLZ-211 as an oncofetal gene [23]. In the present study, we verify the utility of CRISPR/Cas9 system in mechanistically studying lncRNAs by deleting UCA1, and verify UCA1 as an onco-lncRNA promoting bladder cancer progression. Further, the clinical significance of urinary UCA1 is evaluated by a meta-analysis.

## RESULTS

### Inhibition of UCA1 expression by CRISPR/Cas9

**Figure 1:**
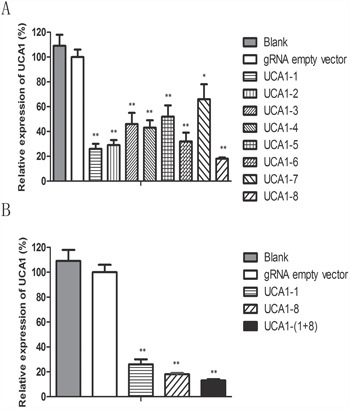
The repression of UCA1 expression by CRISPR/Cas9 **A.** The CRISPR/Cas9 plasmids were transfected into 5637 cells. After 48 h transfection, the cells were collected and the level of UCA1 was measured by qRT-PCR. gRNA named as UCA1-1 targeting UCA1 exon 1 and gRNA named as UCA1-8 targeting promoter region of UCA1 displayed the most significant suppression on UCA1 level. **B.** And the co-transfection of CRISPR/Cas9-UCA1-1 and -UCA1-8 produced synergistic inhibition on UCA1 level. (*P <0.05, **P< 0.01).

In our previous study [24], we found that UCA1 was expressed in both 5637 and T24 bladder cancer cell lines, and UCA1 level in 5637 cells was higher than that in T24 cells. Therefore, we first chose 5637 cell line to examine the knocking down effect of CRISPR/Cas9 on UCA1 expression level. A total of 8 gRNAs targeting UCA1 were designed and subcloned into CRISPR plasmid. Their effects on UCA1 level were determined by quantitative RT-PCR after transfection of each CRISPR/Cas9, respectively, into cells. All of the 8 gRNAs exhibited knocking down effect on UCA1 level in 5637 cells (Figure 1A), among which UCA1-1 gRNA targeting UCA1 exon 1 and UCA1-8 gRNA targeting UCA1 promoter presented most significant inhibition on UCA1 expression, as illustrated by about 85% and 80% of reduction, respectively, in UCA1 level relative to the negative control. We further co-transfected CRISPR/Cas9-UCA1-1 and -UCA1-8 into 5637 cells and observed synergistic inhibition on UCA1 expression (Figure 1B). Therefore, we obtained at least two effective gRNAs that highly suppressed UCA1, and we found that multiple gRNAs could be simultaneously used to gain increased deletion efficiency.

**Figure 2:**
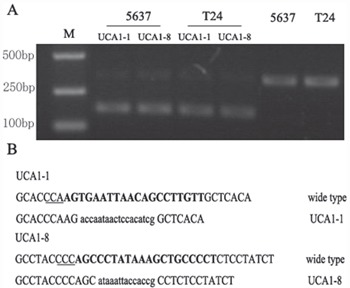
Targeted disruption of DNA sequences *in vitro* **A.** The T7 endonuclease 1 (T7E1) assay was conducted with DNA isolated from cells transfected withgRNA empty vector or CRISPR/Cas9-UCA1 plasmids. The smaller digested fragment was only presented in cells transfected with CRISPR/Cas9-UCA1(1+8) plasmids. M=DNA marker. **B.** DNA sequencing results of the sites targeted by CRISPR/Cas9-UCA1. The target sequences of CRISPR/Cas9-UCA1 were indicated in bold, and the protospacer adjacent motif (PAM) sequences were highlighted as underlined text. The deletions and insertions are presented as lower case letters.

Next, we verified that the knocking down effect of our CRISPR/Cas9 system was resulted from the specific cleavage of DNA coding for UCA1. The genomic DNA isolated from 5637 and T24 bladder cancer cells transfected with CRISPR/Cas 9-UCA1-1 or UCA1-8 was analyzed using T7 endonuclease 1 assays and DNA sequencing. The results showed the presence of target-specific cleavage (Figure 2A) and mutagenesis (Figure 2B) of DNA in 5637 and T24 cells transfected with CRISPR/Cas9-UCA1-1/UCA1-8. These mutation patterns were results of DNA repair in the non-homologous end joining (NHEJ) pathway and genome editing, which strongly suggested that our CRISPR/Cas9-UCA1 gRNA systems generated double-stranded DNA breaks at the specific DNA target sites.

### Downregulation of UCA1 attenuated malignant phenotypes of bladder cancer cells

**Figure 3:**
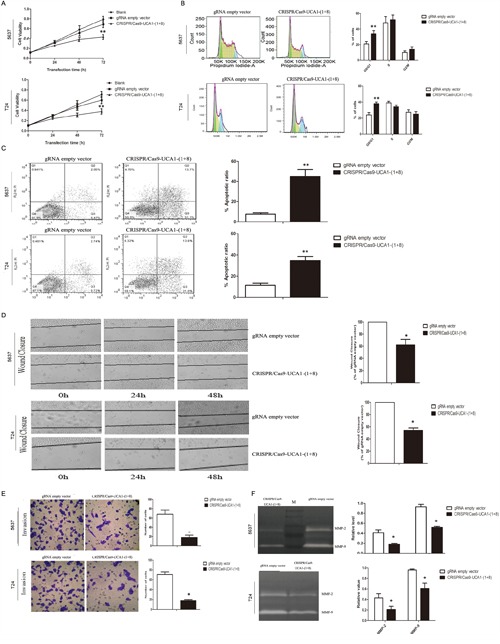
Effects of UCA1 downregulation on the proliferation, migration and invasion of bladder cancer cells *in vitro* **A.** Cell viability was assayed at 0 h, 24 h, 48 h and 72 h post-transfection timepoints by MTT assay. gRNA empty vector did not impact cell viability compared to blank control, while CRISPR/Cas9-UCA1(1+8) plasmids impaired cell viability which became significant after 72 h transfection. **B.** Knocking down of UCA1 arrested cell cycle at G1 phase. **C.** UCA1 downregulation induced early and late apoptosis of bladder cancer cells. **D.** Suppression of UCA1 retarded wound healing of bladder cancer cells. **E.** Transwell assays showed that reduction of *in vitro* invasion of CRISPR/Cas9-UCA1(1+8) plasmids-transfected cells (200 × magnification). **F.** Inhibition of the release of MMP-2/9 was observed in CRISPR/Cas9-UCA1(1+8) plasmids-transfected cells. M=protein marker. (*P <0.05, **P< 0.01).

To investigate the functional roles of UCA1 in bladder cancer, we observed the changes of cell phenotypes caused by UCA1 downregulation via CRISPR/Cas 9-UCA1-1 or UCA1-8 transfection. MTT assay showed that CRISPR/Cas9-UCA1-(1+8) transfection impaired viability of 5637 and T24 bladder cancer cells, which became apparent after 48 h- transfection (Figure 3A). Cell cycle analysis showed that UCA1 downmodulation mainly arrested cell cycle at G0/G1 phase (Figure 3B). Apoptosis analysis by flow cytometry displayed that suppression of UCA1 caused significant early and late apoptosis (Figure 3C). Downregulation of UCA1 also inhibited cell mobility as shown by retard of wound closure (Figure 3D), and cell invasion as shown by results of transwell assay (Figure 3E) and gelatin zymography (Figure 3F). These *in vitro* results indicated that UCA1 acted as an oncogenic lncRNA in bladder cancer.

### Inhibition of UCA1 suppressed growth of xenografts in nude mice

**Figure 4:**
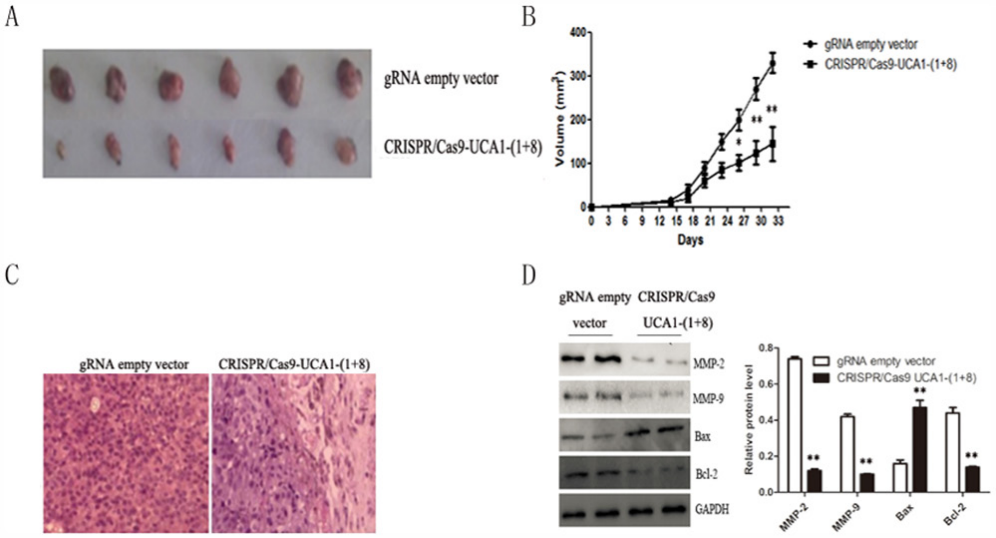
Knocking down of UCA1 inhibited bladder cancer cell proliferation *in vivo* The 5637 cells transfected with CRISPR/Cas9-UCA1-(1+8) or gRNA empty vector were subcutaneously inoculated into nude mice. **A.** Mice were sacrificed and the tumors were isolated after 33 days of inoculation. The growth of CRISPR/Cas9-UCA1-(1+8)-transfected cells was retarded Compared to the gRNA empty vector-transfected cells. **B.** The tumor size were smaller in mice inoculated with CRISPR/Cas9-UCA1-(1+8)-transfected cells compared to negative control. And the difference in tumor size became significant after 27 days of inoculation. **C.** H&E staining results of xenograft tumor tissues (40× magnification) showed that cell proliferation was decreased in xenografts formed by CRISPR/Cas9-UCA1-transfected cells **D.** The expression of MMP-2, MMP-9, Bcl-2, and Bax were determined by western blot which were consistent with that of *in vitro* results. (*P < 0.05, ** *P* < 0.01).

To confirm the consequences of UCA1 downregulation by CRISPR/Cas9-UCA1-(1+8) in cell models, 5637 cells transfected with CRISPR/Cas9-UCA1-(1+8) or gRNA empty vector were subcutaneously inoculated into nude mice. As shown in Figure 4A and 4B, xenografts derived from UCA1-knocking down cells grew more slowly in comparison with the negative control cells. Immunostaining showed that the cell proliferation was significantly decreased in tumors formed by UCA1-suppressed cells (Figure 4C). As shown in Figure 4D, western blot analysis revealed that pro-invasion proteins MMP2 and MMP9 and anti-apoptotic Bcl-2 were significantly decreased while pro-apoptotic Bax was substantially increased in xenografts formed from UCA1-suppressed cells.

### Meta-analysis

**Figure 5:**
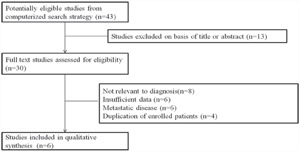
Flow diagram of the publication selected process

The results of literature search were shown in Figure 5. The literature search identified 43 potentially relevant studies; 13 literatures were excluded after screening the title and abstract, for example: they were reviews, meta-analyses, or letters. The full-text studies were retrieved for assessment in detail. 24 were excluded because of various reasons (8 irrelevant to diagnosis, 6 without sufficient data, 6 about metastatic disease, and 4 other studies with duplicate data). Finally, 6 case-control studies [25–30] were included in the meta-analysis

**Figure 6:**
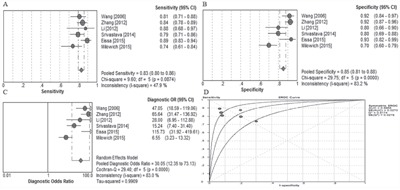
Meta analysis **A.** Forest plots of sensitivity, **B.** specificity, and **C.** summary diagnostic odds ratio (DOR) plots of UCA1 determination in the diagnosis of bladder cancer. **D.** Summary receiver operating characteristic curves for urinary UCA1.

Study characteristics included in the meta-analysis are presented in Table 1. A total of 6 research articles involving 619 bladder cancer patients and 491 healthy people were included in this meta-analysis. The publication year of included article ranged from 2005 to 2015. QUADAS-2 system was used and the results assessment results turned out to be high. A forest plot of the meta-analysis about sensitivity, specificity and area under the curve were shown in Figure 6. A random-effects model was used. The pooled assessment outcomes for 6 studies were as follows: sensitivity, 0.83 (95% CI= 0.80-0.86); specificity, 0.83 (95% CI=0.80-0.86); DOR, 0.83 (95% CI=0.80-0.86); and area under the curve (AUC), 0.83 (95% CI=0.80-0.86), indicating a high diagnostic accuracy of urinary UCA1 for bladder cancer.

**Table 1:** Characteristics of studies included in the meta-analysis

First author	Year	Country	Cancer type	Method	Treat	Control	Sample
Wang [25]	2006	China	Bladder cancer	PCR	94	85	urine
Zhang [26]	2012	China	Bladder cancer	PCR	180	144	urine
Li [27]	2012	China	Bladder cancer	PCR	24	50	urine
Srivastava [28]	2014	India	Bladder cancer	PCR	117	74	urine
Eissa [29]	2015	Egypt	Bladder cancer	PCR	139	45	urine
Milowich [30]	2015	Belgium	Bladder cancer	PCR	65	93	urine

## DISCUSSION

Although human genome is pervasively transcribed, only about 2% of the genome encode protein [31]. After the discovery of ncRNA such as lncRNA and microRNA, increasing researches report dysregulated lncRNA expression among various cancers and suggest that lncRNA is closely related to tumorigenesis, metastasis or recurrence either as oncogenes or tumor suppressors. Furthermore, lncRNAs may serve as independent biomarkers for cancer diagnosis and prognosis [32–35]. However, biological information about lncRNAs is far from adequate compared to that of protein-coding genes. The major hurdle for functional research of lncRNAs is the lack of effective tools to inhibit their transcription. Many lncRNAs are localized in the nucleus [36], difficult to be knocked down by RNAi [37]. Thus, techniques of genetic editing at the genomic level provide better alternatives because they target the genome DNA. Development of sequence-specific nucleases like zinc finger nuclease (ZFNs) [38] and transcription activation-like element nuclease (TALENs) [39] have been available for this purpose, including. Recently, a novel breakthrough technology for genetic engineering called clustered regularly interspaced short palindromic repeats (CRISPR)/CRISPR-associated (Cas) system with high efficiency of gene editing is emerging [40, 41]. It is easily generated since the realization of the technique only requires changes of the sequence of the guide RNA (gRNA) subcloning into specific CRISPR/Cas9 system. Furthermore, multiplexing capability of CRISPR/Cas9 makes it possible to target multiple genes simultaneously [42]. Since the first report in the bacterial system [43], a large number of articles have been published [44, 45, 46, 47]. Our previous studies have harnessed the CRISPR/Cas9 system to disrupt the hepatitis B virus and HPV [10, 11]. However, most of the studies are focused on protein coding genes. Although two reports indicated the feasibility of knocking out non-coding genes by the CRISPR/Cas9 system in human cells [48, 49], none of them use a pool of gRNAs targeting lncRNA. Here, we explore the applicability of combined CRISPR/Cas9 systems to target lncRNA UCA1 for its functional studies *in vitro* and *in vivo*.

**Figure 7:**
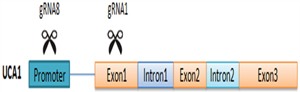
Strategy for targeting UCA1 with co-transfection of CRISPR/Cas9-UCA1-1/8 plasmids

To investigate the functions of lncRNA UCA1 in bladder cancer, we designed eight gRNAs targeting the promoter or the exon of UCA1, and we obtained two gRNAs with highly suppressive activity for this research. Strategy for targeting UCA1 with combination of the two gRNAs was shown in Figure 7. Transfection of CRISPR/Cas9-UCA1 systems resulted in targeted genome-specific DNA cleavage. Combined use of CRISPR/Cas9-UCA1-1 and –UCA1-8 suppressed UCA1, inhibited cell proliferation, migration and invasion, and induced cell cycle arrest and apoptosis, which was verified by *in vivo* experiments. Thus, we showed the utility of CRISPR/Cas9 in the modulation of lncRNA and verified the oncogenic role of UCA1 in bladder cancer. And the influence of CIRSPR/Cas9- UCA1 on the malignant phenotypes of 5637 and T24 cells were almost the same which was similar to the published data as well [23, 24, 50, 51, 52]. Actually, the function of UCA1 in bladder cancer cells were consistent that it promoted cell cycle progression, apoptosis inhibition, and MMPs enhancement [24, 50].

Bladder cancer is one of the most common malignant tumors in the world, with particularly high incidence in China [53, 54]. Although cystoscopy combined with urinary cytology increase the early diagnosis of bladder cancer, many patients are diagnosed at the advanced stage and have poor prognosis [55, 56]. Thus, new markers for screening, early diagnosis, and surveillance for recurrent lesions, with the ultimate aim to improve clinical management of patients, are still in urgent need [57]. We recruited a total of 6 independent studies investigating the urine UCA1 level in the meta-analysis. Our analysis indicated that urine UCA1 as a non-invasive diagnostic marker for bladder cancer. In conclusion, our study first reported that CRISPR/Cas9 could be used to effectively suppress lncRNA level, and highlighted that UCA1 as an oncogene of bladder cancer.

## MATERIALS AND METHODS

### *In vitro* experiments

### Plasmids

The hCas9 expression vector (plasmid 44758) and gRNA cloning vectors (plasmid 41824) were obtained from Addgene (Cambridge, MA, USA). The plasmids were prepared by using the Qiagen Endofree Plasmid Kit (Qiagen, Hilden, Germany).

### Design and cloning of UCA1-specific gRNA

gRNA expression plasmids were constructed according to manufacturer's protocol [46] and detailed BLAST searches of the human and murine genomes were conducted to identify potential off-target binding of UCA1 gRNAs. To assess the utility of UCA1-targeting gRNAs, 8 sets of oligonucleotides (Table 2) were designed to target the complete genomic UCA1. All oligonucleotides were synthesized and purified by Sangon Biotech Co (Shanghai, China). Briefly, to prepare a 100-bp double-stranded DNA insert fragment containing the target sequence (20 bp) and a protospacer-adjacent motif sequence, we used a set of oligonucleotides and generated the fragment using T4 PNK (NEB, Ipswich, MA, USA). The double-stranded DNA fragment was purified and inserted into the BbsI site of a gRNA cloning vector with T4 DNA ligase (NEB, Ipswich, MA, USA).

**Table 2:** The sequences and location of gRNA

Name	Genomic target	Target location
UCA1-1	CACCGTGGATCTCTTCACGGAATG CACCTAGAGAAGTGCCTTACCAAA	89-109
UCA1-2	CACCGTCTGAAAAGAGAGTCAGCGA CAGACTTTTCTCTCAGTCGCTCAAA	114-134
UCA1-3	CACCGTGTGCTATAAATGACCGGGT CACACGATATTTACTGGCCCACAAA	189-209
UCA1-4	CACCGGGGGACTCCTTCGTGAGAC CCCCCTGAGGAAGCACTCTGCAAA	201-221
UCA1-5	CACCGCTTCGGGTAACTCTTACGG CGAAGCCCATTGAGAATGCCCAAA	265-285
UCA1-6	CACCGCGTAAGAGTTACCCGAAGCT CGCATTCTCAATGGGCTTCGACAAA	289-309
UCA1-7	CACCGTGCTATAAATGACCGGGTA CACGATATTTACTGGCCCATCAAA	356-376
UCA1-8	CACCGAGGGGCAGCTTTATAGGGCT CTCCCCGTCGAAATATCCCGACAAA	24-44

### Cell culture and transfection

5637 and T24 bladder cancer cell lines were obtained from Chinese Type Culture Collection of Chinese Academy of Sciences, maintained in our laboratory and routinely cultured in RPMI 1640 medium (Invitrogen), supplemented with 10% fetal bovine serum at 37°C in a humidified atmosphere containing 5% CO2. All experiments were performed using the log phase cells.

The 5637 cells and T24 cells were seeded in 6-well plates 24 h prior to transfection. Polyethylenimine was used to transfect cells with 1μg of the hCas9 expression vector and 1μg of the gRNA expression vector. The control group was transfected with no vector, and the gRNA-empty vector group was transfected with 1μg of hCas9 expression vector and 1μg of gRNA-empty vector using Lipofectamine2000 (Invitrogen, Carlsbad, CA, USA) according to the manufacturer's instructions.

### T7 endonuclease 1 assay

To confirm that DNA cleavage and targeted sequence disruption occurred at the intended site, a mismatch-sensitive T7 endonuclease 1 assay (NEBIpswich, MA, USA) was performed. The DNA region encompassing the S- and X-target site was amplified under standard conditions using the following primers: S1 forward: 5′-CCTTAACTAATTAACCCACC-3′and reverse: 5′-AAGAGAGTCAGCGAAGGGAG-3′. The PCR products were subjected to heteroduplex formation after denaturing 200 ng of amplified DNA at 95°C for 5 min followed by slow cooling to 35°C. The samples were treated with 5 U of T7 endonuclease 1 in 1 × NEB Buffer 2, incubated at 37°C for 30 min and then analyzed by agarose gel electrophoresis. Tanon electrophoretical software (Tanon Science & Technology Co., Ltd., Shanghai, China) was used to measure band intensities, and the targeted disruption was observed according to the previous description by Guschin et al [58].

### RNA isolation and quantitative RT-PCR

Total RNA was extracted from bladder cell lines using TRIzol reagent (Invitrogen Carlsbad, CA, USA) according to the manufacturer's instructions. The expression levels of UCA1 were determined by quantitative RT-PCR using a SYBR Premix Ex Tap II kit (Takara, Dalian, China) on a CFX96 real-time PCR System (Bio-Rad, Hercules, CA, USA), and the results were normalized using β-actin as an internal control.

### Cell proliferation assay

The MTT [3-(4,5-dimethylthiazol-2-yl)-2,5-diphenyltetrazolium bromide] (Sigma-Aldrich, MO, USA) colorimetric assay was used to validate the cell viability [59]. 15,000 cells per well were seeded in 96-well plates on the day prior to transfection. Groups of replicates included cells that were untransfected, CRISPR+Cas9+gRNA empty vector transfected, or transfected with CRISPR+Cas9+UCA1 gRNA. Cell viability was assessed 3 days after transfection by adding 20 μl of MTT (5 mg/ml) to each well, and incubating at 37°C for 1 h. Metabolism of the MTT to form the blue formazan was determined by measuring the ratio of optical density at a wavelength of 570 nm, to the background at 690 nm.

### Apoptosis analysis

Cells were distributed on a 6-well plate at a density of 5 x 10^5^ per well. After transfection for 48 h, cells were harvested and washed with PBS three times. Then the cell apoptosis was detected by Annexin V/FITC and propidium iodide (PI) binding assay according to the manufacturer's instructions (BD Biosciences, Franklin Lakes, NJ, USA). The mixed solution was gently shaken and stored away from light at room temperature for 15 min. The stained cells were analyzed directly by flow cytometry using Cell Quest software (BD Biosciences, Franklin Lakes, NJ, USA).

### Cell cycle analysis

Cells transfected with CRISPR/Cas9 vectors were washed with PBS and fixed with 75% ethanol overnight at -20°C. Cells were resuspended in PBS and treated with RNase for 30 min at room temperature. The cell nucleus was stained with propidium iodide (Sigma, Saint Louis, MO, USA) and incubated for 20 min in the dark. Cell cycle phases were determined by flow cytometry (BD Biosciences, Franklin Lakes, NJ, USA) and analyzed with FlowJO 7.6 program.

### Wound healing assay

Cell migration assay was performed using 12-well plates as previously described [46]. Cells were seeded into 12-well plates (1 x 10^5^ cells/ml) and grown to 80-90% confluence for the experiment. Cells were scraped with 200 μl sterile pipette tip to create a scratch. They were washed twice with PBS to remove cellular debris and then replaced with complete 1640 medium. Cells were transfected with CRISPR/Cas9 and incubated for 48 h. Cell migration into the wound area was photographed at the timepoints of 0 h, 24h, 48 h, respectively, for the image analysis of each treatment. The wound healing level was determined using a Hewlett-Packard scanner and NIH Image software (Image J).

### Cell migration/invasion assay

The invasive behavior of 5637 and T24 cells was tested using cell invasion chamber kit (BD Bioscience, San Jose, CA, USA). Cells were re-suspended in a serum-free 1640 medium (5x10^4^ cells/200 μl). Cells were seeded onto the upper chamber of Matrigel-coated filter, and 500 μl of RPMI 1640 medium containing 10% FBS was added in the lower chamber. After 48 h incubation, the non-invading cells were removed from the upper surface of the filter membrane. The migrated cells on the lower surface of the filter membrane were stained with crystal violet for 1 h and rinsed with PBS. The amount of invading cells on the lower surface of filter membrane was determined using a light microscope and NIH Image software (Image J).

### Gelatin zymography

MMP-2/9 activity was determined by gelatin zymography. Briefly, cells were seeded (1 x 10^5^ cells/well) and allowed to grow to confluence for 24 h and maintained in 1640 medium with 10% FBS. The cells were washed with PBS and transfected with CRISPR/Cas9 systems for 48 h. The supernatant was then collected and mixed with loading buffer, then electrophoresed in 10 % polyacrylamide gel containing 0.1 % (w/v) gelatin. After the electrophoresis, gel was washed with washing buffer (2.5 % Triton X-100 in dd H_2_O) for 30 min and incubated at room temperature for additional 18 h for the enzymatic reaction of MMPs in zymography reaction buffer (200 mM NaCI, 10 mM CaCl_2_, 50 mM Tris-HCI, PH 7.4). The gel was then stained with Coomassie blue R-250(0.125% Coomassie blue R-250, 50% methanol, 10% acetic acid) for 1 h and destained with destaining solution (20 % methanol, 10 % acetic acid, 70 % dd H_2_O) until the clear bands were visualized.

### *In vivo* experiments

Approval for animal experiments was obtained from the institutional animal welfare committee. Pathogen-free male BALB/C nude mice (aged 4-5wk, SPF grade and weighing 18-20g) were purchased from the center of experimental animal, the Academy of Military Medical Science (Beijing, China). The nude mice were caged individually under specific-pathogen free (SPF) conditions. All animal experiments were performed strictly in accordance with the Guide for the Care and Use of Laboratory Animals of the National Institutes of Health. The mice were randomly assigned to the control or experimental group (six mice per group). 5637 cells transfected with gRNA empty vector or CRIPSR/Cas9-UCA1-(1+8) were harvested and injected subcutaneously into each mouse (2x10^6^/0.2 ml). Tumor volume was estimated every three days by the formula: 0.5x length x width^2^. All mice were sacrificed after 33 days. Tumor tissues were excised, paraffin-embedded, formalin-fixed and H&E stained, and were further analyzed by western blot.

### Western blot

Total protein was extracted with RIPA buffer (50 mM Tris-HCl [PH 7.5]), 150nM NaCl, 1% Triton X-100, 0.5% Na-deoxycholatc) containing protease inhibitiors. 30 μg samples of the lysates were separated on 8%-12% SDS-PAGE gels and transferred to PVDF membranes. The membranes were incubated with primary antibodies including anti-human MMP2 and MMP9 (Abcam, Cambridge, MA, USA; 1:500), Bax and Bcl-2 (Cell Signaling Technology, Beverly, MA, USA; 1:500) overnight at 4°C followed by incubation with a HRP-conjugated secondary antibody. Finally, the blots were detected using ECL substrate.

### Clinical study

#### Meta analysis

#### Study strategy

This meta-analysis was conducted in accordance with the Preferred Reporting Items for Systematic Reviews and Meta-analyses (PRISMA) guidelines [60]. A prespecified protocol that included the data sources, search strategy, inclusion/exclusion criteria for the articles, and analysis methods was developed before the beginning of this study.

We followed the Cochrane Handbook for Systematic Reviews of Diagnostic Test Accuracy and the Meta-analysis Of Observational Studies in Epidemiology (MOOSE) [61]. The identification of relevant studies was conducted in a search of the PubMed, MEDLINE, EMBASE and China National Knowledge Infrastructure (CNKI) databases for the period of Jan 2000 to Dec 2013. The search term was “UCA1”, “long intergenic noncoding RNA”, “noncoding RNA”, “lncRNA”, “lincRNA”, “bladder cancer”, “bladder carcinoma”, “bladder neoplasm”, “prognosis”, “prognostic” Clinical and pathologic variables were also dichotomized. For each variable, summary odds ratios and their 95% confidence intervals were estimated using the inverse variance weighted method. Because the meta-analysis involved expression data assessed by different methods, we used the random-effects model. Forest plots were used to present the results. The meta-analysis was conducted with the use of Meta-Disc 1.4 (Ramon y Cajal Hospital, Madrid, Spain).

#### Study selection

The same two investigators independently assessed all the eligible studies and extracted the data. Studies were considered eligible if they met the following criteria: any type of human bladder cancer was studied; UCA1 expression was determined in human urine using quantitative PCR; If data subsets were published in more than one article, only the most recent one was included. Citations were limited to those published in the English language. If the data could not be extracted or calculated from the original article, the study was excluded. Disagreements were resolved through discussion with Correspondence author.

#### Inclusion and exclusion criteria

The studies qualified to be included must have fulfilled the following criteria: (i) investigated the diagnostic potential of (urine) UCA1 for human bladder cancer, (ii) used the gold standard to confirm the diagnosis of cancer patients, and (iii) provided sufficient data. The studies are excluded if they (i) are obviously not related to our topic; (ii) are in forms of letters, editorials, case reports, or reviews; and (iii) used other types of samples other than urine.

#### Data extraction and quality assessment

Data from all eligible studies were extracted as follows: (i) basic characteristics of publications, including name of first author, year of publication, country of the study, ethnicity of subjects, number of participants, types of disease, and types of samples, and (ii) diagnostic results, including sensitivity, specificity, true positive, false positive, false negative, and true negative, respectively. Quality Assessment of Diagnostic Accuracy Studies 2 (QUADAS-2) was used to evaluate the quality of included publications.

### Statistical analysis

The SPSS 16.0 software system (SPSS, Chicago, IL, USA) was used for statistical analysis. Data are expressed as the mean+-standard error (SD). The differences between groups were analyzed using a Student t test when only 2 groups were compared or one-way analysis of variance when more than 2 groups were compared. Kaplan-Meier method and log-rank test were performed for patients’ survival analysis. All experiments were run in triplicate. P<0.05 was considered statistically significant, and P< 0.01 was considered highly significant.
